# The acceptability of cervical electrical impedance spectroscopy within a multi-modal preterm birth screening package: a mixed methods study

**DOI:** 10.1186/s12884-022-05202-z

**Published:** 2022-12-22

**Authors:** Victoria Stern, Georgina L. Jones, Sarah Senbeto, Dilly Anumba

**Affiliations:** 1 Academic Unit of Reproductive and Developmental Medicine, Department of Oncology and Metabolism, University of Sheffield, Sheffield, UK; 2 School of Social Sciences, Leeds Beckett University, Leeds, UK; 3 Jessop Wing, Sheffield Teaching Hospitals NHS Foundation Trust, Sheffield, UK

**Keywords:** Electrical impedance spectroscopy, Cervix, Screening, Preterm birth, Preterm labour, Acceptability, Maternal experience, Mixed methods, Triangulation

## Abstract

**Background:**

Reducing the rate of preterm birth is a cornerstone of global efforts to address child mortality, however existing screening tests offer imperfect prediction. Cervical electrical impedance spectroscopy (EIS) is a novel technique to quantify the ripening changes which precede labour. Mid-trimester EIS measurements have been shown to accurately predict preterm birth in asymptomatic women. This study aimed to comprehensively evaluate the acceptability of cervical EIS to low and high-risk women as part of a package of screening tests performed during a larger prospective trial.

**Methods:**

In this parallel convergent mixed methods study, 40 women completed questionnaires before and after screening tests (EIS, cervical length measurement and fetal fibronectin quantification). Quantitative outcomes were anxiety levels before and after screening (Spielberger State Trait Anxiety Inventory, STAI-6), pain (Short Form McGill Pain Questionnaire) and ratings of EIS device appearance and test acceptability (visual analogue scales). Twenty-one women (11 high-risk, 10 low-risk) also attended a semi-structured qualitative interview. Interviews were recorded and transcribed, then thematic analysis was performed. A convergence coding matrix was constructed to enable triangulation of quantitative and qualitative results.

**Results:**

High risk women demonstrated a significant reduction in anxiety following screening (mean STAI-6 score 34.5 *vs.* 29.0, *p* = 0.002). A similar trend was observed among low-risk participants. Ratings of pain, EIS device appearance and procedural acceptability did not differ between groups. Mean pain ratings were low (visual analogue scale 0.97 and 1.01), comparing favourably to published evaluations of conventional screening tests. Qualitative analysis provided insight into both the physical consequences and emotional experiences of screening. Additional determinants of the screening experience included device design, pre-existing perspectives on intimate examination, attitudes to knowledge in pregnancy and interaction with clinical staff. Finally, a range of practical considerations regarding wider use of EIS were identified, with valuable complementary detail regarding acceptability for use in antenatal care.

**Conclusions:**

Cervical EIS is well tolerated and acceptable to both low and high-risk women when performed as part of a multi-modal screening package. These results provide useful insights to inform the design of future study and screening protocols.

**Supplementary Information:**

The online version contains supplementary material available at 10.1186/s12884-022-05202-z.

## Background

Worldwide, preterm birth (PTB) is the leading cause of neonatal mortality [1, 2] with significant long-term morbidity amongst survivors [2]. Prediction and prevention of PTB have repeatedly been identified as key to improving obstetric outcomes [3, 4]. However, the PTB syndrome has diverse aetiologies [5] thus calculating which women will deliver early is challenging. Increasingly global, including UK, guidance recommends that women with prior PTB or late miscarriage undergo transvaginal ultrasound (TVUSS) measurement of cervical length (CL) [6]. Other commentators advocate the addition of quantitative vaginal fetal fibronectin (FFN) estimation in high-risk asymptomatic women. However, both tests have relatively poor predictive performance when applied to an unselected or low risk population [7, 8], and effective universal screening tests prove elusive. The ideal screening programme would offer good predictive accuracy for both high and low risk groups as nulliparous women, in particular, are not well served by existing risk-factor based approaches.

The cervix represents the “final common pathway” in the process of preterm parturition [5] – it must remodel and dilate for PTB to occur, regardless of the initial trigger. It is therefore a logical target for PTB screening. Electrical impedance spectroscopy (EIS) is a technique with proven ability to interrogate tissue structure [9–12], originally used in the detection of pre-malignant changes within the cervix [9]. A recent prospective study of asymptomatic women confirmed that mid-gestation cervical EIS measurements can accurately predict spontaneous PTB before 37 weeks [13] and has potential for incorporation into existing risk prediction algorithms [14]. It is plausible that using a multi-modal package of screening tests might optimise PTB prediction and enhance predictive accuracy in both low and high risk groups. Further studies are necessary to confirm the benefit of incorporating EIS into existing screening protocols, but it is also essential to confirm that it is acceptable to pregnant women. We therefore conducted a parallel convergent, mixed methods study to systematically evaluate the acceptability of EIS to women at both high and low risk of preterm birth. This sub-study was nested within a larger prospective trial examining the predictive accuracy of EIS [13].

No previous research has examined patients’ experiences of undergoing EIS measurements. Furthermore, the literature regarding women’s perspectives on PTB screening is relatively sparse. Studies are predominantly quantitative, with questionnaires employed to examine factors such as pain, anxiety, and embarrassment during CL scans [15–19] and anxiety associated with FFN testing [18, 20]. More recently, the impact of the Quantitative Instrument for the Prediction of Preterm Birth application (QUiPP app) (which combines obstetric history, CL and FFN to estimate PTB risk) has also been assessed via questionnaire [21].

Five qualitative studies have considered screening as a factor in the experiences of women at risk of PTB; these have studied high-risk asymptomatic women [22], those with symptoms of preterm labour [23–25] or a mixture of the two [26]. All employed semi-structured interviews either individually or via focus groups. Three focused on women’s experiences of antenatal care, via PTB clinic attendance [22] or during symptomatic presentations [23, 24]. Two specifically examined women’s decision-making during episodes of threatened preterm labour [25, 26].

No research has been identified which uses mixed methods to synthesize both quantitative and qualitative data. Such a technique can be advantageous in providing a comprehensive view of patient experience. Quantitative instruments such as questionnaires are practical to apply to larger groups, thus may capture a larger range of views—this likely explains the dominance of this approach in existing literature. When appropriately designed, they should have external validity, allowing more confident generalisation of their results. However, qualitative interviews have the ability to provide context, explanatory detail and illustration and may help explain why particular patterns of quantitative response are observed, Furthermore, triangulation of quantitative and qualitative datasets can enhance validity, allowing areas of convergence, dissonance and silence to be highlighted [27, 28]. High convergence between datasets offers corroboration of their findings, whereas identification of dissonance prompts careful examination to understand the reasons for any inconsistency. Silence (when one methodology identifies themes on which the other is silent) may be expected to a degree as quantitative and qualitative approaches often examine different aspects of a research question, but unexpected areas of silence may provide a prompt for further investigation[28]. We therefore aimed to employ validated quantitative measures of pain and anxiety to examine women’s experiences of EIS (allowing comparison to existing PTB screening literature) but also to conduct semi-structured qualitative interviews to obtain further information regarding women’s EIS and PTB screening experiences in general (to enhance and explain our quantitative findings). Given the novel nature of EIS, the ability of qualitative interviews to elicit detailed accounts of women’s experiences of testing was important to maximise understanding of how the test may impact patients.

## Methods

Ethical approval for this study was provided by the Yorkshire and Humber National Research Service Ethics Committee (13/YH/0167).

### Setting

**Fig. 1:**
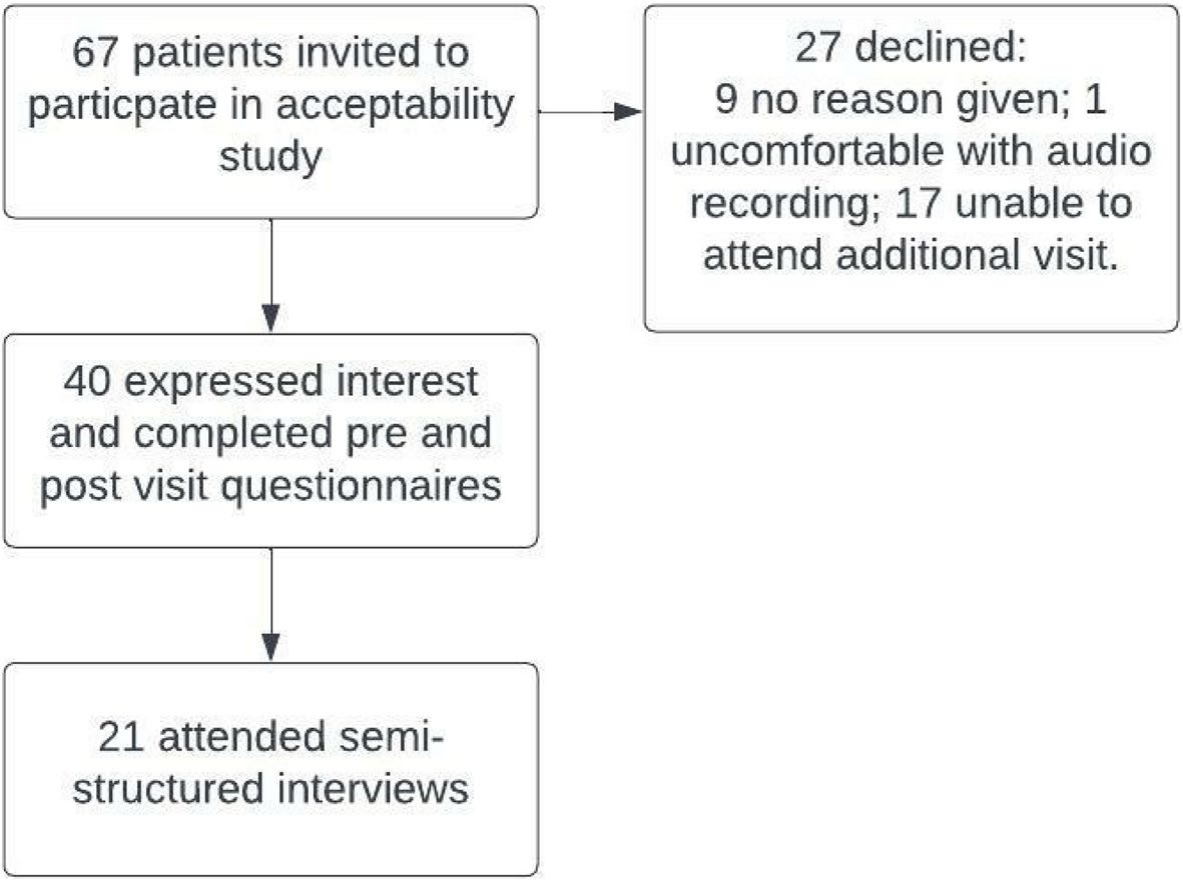
Flow diagram of recruitment to acceptability study

Women received verbal and written information about the main EIS study during booking appointments at the Jessop Wing, Sheffield (a large teaching hospital) via the research midwife (SS) or clinical research fellow (VS). They were eligible for inclusion if aged over sixteen, with good comprehension of English and were carrying a singleton pregnancy, with no evidence of fetal anomaly. Non-English speakers, women with multiple pregnancies or those with factors which precluded accurate EIS measurements (recent abnormal smear test, active cervical infection or bleeding) were excluded. Recruitment took place between January 2014 and April 2016. Low-risk women (LRW) were primiparous or multiparous with no risk factors for PTB. High-risk women (HRW) had a history of one or more previous PTB and/or late miscarriages. If interested, they were later contacted to confirm recruitment. Women were asked their preferred method of communication (phone call, text message or email) and follow up contact was made the week after initial approach, with more time for consideration given if requested. Travel and parking expenses were renumerated but no other incentive to participate was provided. LRW attended one research visit at 20–22 weeks, HRW again at 26–28 weeks. The patient information sheet and verbal counselling provided to prospective recruits made it clear that both clinicians and participants would be blinded to their EIS results during the study. They were informed that EIS showed predictive promise during a pilot study and that the purpose of the main study was to assess whether this accuracy could be replicated on a larger scale, therefore it would not influence their clinical care. It was also made clear to women what existing clinical protocols and national guidance advised with respect to preterm birth screening in their individual case (i.e. serial cervical length scans with consideration of prophylactic treatment if indicated for those with risk factors for preterm birth, no screening or prophylactic therapy for those without risk factors). A summary of the recruitment process is provided in Fig. 1.

At the main research visit women underwent a series of tests: an initial speculum examination (when swabs were taken for infection screening and FFN quantification, then EIS measurements were obtained) followed by a CL scan. The EIS probe emits a ‘beep’ when taking a reading, thus women were aware when measurements were being obtained during the speculum examination. Women received results of the CL scan immediately and no information regarding their EIS measurement (as above, all were informed it would not be possible to interpret EIS results during the study). Women either received their FFN result during the research appointment or were contacted shortly afterwards. Appropriate follow up was arranged for those with abnormal results. As a minimum, women were offered to attend for a further cervical length scan to ensure no progressive shortening was evident; the differential evidence base for the interpretation of CL and FFN in high risk and low risk women was re-iterated and follow up individualised depending on obstetric history and the wishes of the woman. All results were explained and treatment arranged if necessary. Care was taken when counselling low risk women to emphasize the very limited evidence base for prophylactic therapy in their group (including the absence of evidence for treating LRW on the basis of FFN results; that UK guidance did not advise cerclage for cervical shortening in LRW or the use of progesterone outside a research trial, although American and Australian guidance at the time advised considering progesterone if cervical length less than 20 mm). The same clinician (VS) conducted all study visits.

### Data collection

Before the first study visit, forty women were also invited to participate in the acceptability study. This group was identified via purposive convenience sampling over 12 months spanning the mid-point of recruitment to the clinical trial (November 2014 to October 2015) – this ensured the study procedures were well embedded and operator experience with the EIS probe was high. Those expressing interest completed two questionnaires: a short pre-visit anxiety rating (administered immediately before the three predictive tests) and a longer post-visit questionnaire (administered at the end of the study visit once all tests had been performed). Introductory text explained that the specific purpose of the questions was to assess the acceptability of EIS. Discrimination of the timing of EIS measurements was possible due to both the sensation of contact with the cervix and due to the ‘beeping’ noise made by the EIS probe during clinical measurement. After expression of interest, women were later contacted by a research midwife to confirm participation in the interview stage. Twenty-one women consented and attended an interview. Once recruitment to the interview phase was complete, no further questionnaire data was collected. Where possible, interviews were arranged within four weeks of the main study visit. They were conducted by a research midwife (SS) with training and experience of qualitative interviewing. The use of purposive sampling aimed to ensure a balance of low and high-risk participants, with a range of ages, ethnicities, socio-economic statuses and varied obstetric histories. Recruitment to interview continued until saturation of themes was achieved.

Mixed methods research encompasses a broad range of study designs, but typically incorporates both quantitative and qualitative methodologies. However, each aspect may be afforded different priority and methods may be performed in sequence or concurrently, depending on the desired outcome [28]. Our convergent parallel design aimed to afford equal weight to both datasets, with the intention of producing an integrated summary which captured the strengths of both quantitative and qualitative approaches.

### Quantitative data collection

The pre and post-visit questionnaires were designed to assess women’s anxiety before and after tests; any pain experienced during EIS measurements; women’s views of the probe design and overall acceptability of the EIS test. In order to assess anxiety, the six-question short form of the Spielberger State-Trait Anxiety Inventory (STAI-6) was used [29]. This abbreviated version of the full, forty-question STAI has been validated for use in the perinatal period and has high internal consistency and reliability [30]. Given the potential association between maternal stress and preterm birth [31], it was important to evaluate whether undergoing EIS measurements adversely affected anxiety levels.

Pain during EIS measurement was assessed using the short form of the McGill pain questionnaire. This provides a multidimensional measure of pain which has previously been validated in obstetric patients [32]. It includes two measures of pain intensity: the visual analogue scale (VAS) and the Present Pain Intensity (PPI) plus a Pain Rating Index (PRI) designed to assess the qualities of any pain experienced. Finally, women rated their perception of the appearance of the EIS device and the overall acceptability of the procedure using a ten-point VAS. They were asked to proffer their recommended changes to the testing procedure and the potential acceptability of the procedure for use in routine antenatal care. The ten-point VAS is widely used and validated for the assessment of pain [33, 34] but has also been employed and validated more broadly to evaluate mood [35, 36] and valuation of other health states [37].

### Qualitative data collection

The semi-structured interview schedule (Additional file 1) was designed collaboratively by the interdisciplinary research team (VS, SS, GJ and DA). It consisted of nine open and two closed questions designed to elicit women’s experiences of attending the visit and undergoing tests. The schedule provided a guide, however the interviewer varied the order and structure of the questions as she deemed appropriate, and followed other lines of enquiry if topics of interest arose. Use of a semi-structured approach enabled key objectives to be achieved (obtaining a detailed account of women’s experiences of the tests) whilst allowing flexibility to explore themes which the women themselves might introduce.

The interviewer (SS) was not involved in the clinical care of the women. This neutrality was important to enable participants to reflect freely on their experience without inhibitions or fear of impacting their care. Women chose the location of their interview (at home or in the university research department) and two patients were interviewed during an inpatient antenatal admission (in private side rooms). Interviews lasted 30 min on average and were audiotaped and transcribed verbatim later. Participants provided separate written informed consent for the qualitative study.

### Data analysis

Given the lack of prior studies of EIS acceptability, an exploratory, pragmatic approach, grounded in the lived experiences of our participants felt most appropriate. Pragmatism as a perspective focuses on the cyclical interaction of human beliefs and actions in shaping experience [38]. It enables a technical approach to be taken, in which methods are selected due to their ability to best answer a research question, rather than to fit in with a particular epistemological philosophy [38, 39] and navigates the tension potentially encountered when trying to combine what might otherwise be viewed as conflicting quantitative and qualitative methodologies.

Questionnaire data were analysed using the relevant scoring algorithms and descriptive statistics calculated. Mean pain scores (McGill VAS and PRI), mean anxiety scores before and after testing (STAI-6), mean change in STAI-6 scores, and mean acceptability and device design ratings (both VAS) for high and low risk groups were then compared. The proportion of high and low risk women with high anxiety scores were also compared as were ‘within-group’ pre and post visit scores. Normality of score distribution was assessed via the Shapiro–Wilk test. Mann Whitney U tests were performed to compare nonparametric data whilst independent and paired T tests were performed to compare normally distributed scores. Fisher’s exact test was employed for the comparison of categorical outcomes. Data analysis was performed by VS using SPSS (IBM Corp. Released 2017. IBM SPSS Statistics for Windows, Version 22.0. Armonk, NY: IBM Corp). Application of a Bonferroni correction yielded an adjusted alpha level of 0.005 (0.05/10).

Qualitative analysis proceeded as follows: The first three interviews were transcribed by the interviewer (SS) and the remainder by a research assistant with experience in transcribing qualitative interviews. All transcripts were uploaded to NVivo 10 (QSR International: Burlington MA) and checked for accuracy compared to the audio recordings and any notes taken by the interviewer (VS). The first three interviews (of two HRW and one LRW) were reviewed by three researchers (VS, GJ and SS). Inductive thematic analysis (TA) (following the five-step process described by Braun and Clarke [40]) was employed to develop an initial coding framework, which was continually reviewed during analysis of the remaining interviews. Briefly, this involved familiarisation with the data; generating initial codes; searching for themes; reviewing, defining and naming themes; and producing an overall synthesis, including detailed examples, to interpret and make sense of the data [40]. For the purposes of this study, which aimed to explore women’s experiences of undergoing a novel screening test, the ability of TA to “describe the data set in rich detail” [40] and interpret identified patterns in the context of the overall research question was particularly apposite.

Themes were inductively defined from the raw data through exploration without any predetermined classification where possible. A quarter of the interviews were coded by two researchers (VS and SS) to enable ongoing comparison and refinement of the coding structure, and potential themes were discussed amongst the research team as analysis progressed to maintain reflexivity. Whilst formal assessment of inter-coder reliability is not a pre-requisite for thematic analysis, this comparison of ideas and ongoing dialogue between members of the research team ensured a wide and inclusive approach and was maintained during initial coding.

Following analysis of the two datasets, a mixed methods matrix was constructed (summarising the results of participants with paired data). This enabled systematic comparison of qualitative and quantitative information, specifically looking for areas of convergence, dissonance, silence or complementarity within cases [27]. A convergence coding matrix was also constructed (similar to that advocated by Farmer et al [41]. although generated by a single researcher, VS) to summarise the results of both study components and the triangulation process in a single location (Additional file 2). This enabled the overall questionnaire results to be synthesized with the SSI themes, in addition to the within-case triangulation generated by the matrix.

## Results

### Quantitative survey

Overall questionnaire results are summarised in Table 1.

**Table 1:** Results of quantitative survey

Survey Domain		High Risk Women (*n* = 20)	Low Risk Women (*n* = 20)	*P* value (HR vs LR)
Anxiety				
STAI-6 results	Mean pre-test score (SD)	34.48 (12.72)	29.98 (8.98)	0.204
	Mean post-test score (SD) P value pre *vs.* post test	28.98 (10.20) 0.002	27.50 (9.48) 0.018	0.881
	Mean difference	-5.55 (-20 to 0)	-3.22 (-13 to + 27)	0.628
	Pre-test score ≥ 39	6/20 (30%)	4/20 (20%)	0.273
	Post-test score ≥ 39	5/20 (25%)	2/20 (10%)	0.202
Pain/discomfort				
SF-McGill VAS	Mean VAS score (range)	0.97 (0–3.2)	1.01 (0–3.1)	0.935
SF-McGill PPI	0 – no pain	7/20 (35%)	9/20 (45%)	
	1 – mild pain	11/20 (55%)	9/20 (45%)	
	2 – discomforting	2/20 (10%)	2/20 (10%)	
SF-McGill PRI	Mean Sensory PRI score (range)	1.25 (0–3)	1.60 (0–5)	0.448
	Mean Affective PRI score (range)	0.10(0–1)	0.05 (0–1)	0.553
EIS probe design rating (0 = not threatening; 5 = neutral; 10 = very threatening)				
	Mean VAS score (range)	1.30 (0–5)	1.35 (0–9)	0.988
Acceptability rating (0 = acceptable; 5 = neutral; 10 = unacceptable)				
Personal acceptability	Mean VAS score (range)	0.55 (0–3)	0.75 (0–5)	0.842
Acceptable for use in antenatal care?	Yes	20/20 (100%)	20/20 (100%)	
	No	0/20 (0%)	0/20 (0%)	

### Anxiety

No significant differences in anxiety scores were observed between high and low risk groups at either timepoint. HRW showed a significant reduction in pre- *vs.* post-test STAI-6 scores; A similar trend was observed amongst LRW but did not reach significance (p = 0.002 and 0.018 respectively, Mann Whitney U). There is no universally accepted threshold which defines the presence of significant anxiety, but it has been suggested that scores of 39–40 represent a higher level [42]. When considering those with STAI scores ≥ 39, higher anxiety levels were more prevalent amongst HRW at both time points. However, the incidence of scores ≥ 39 was lower after screening regardless of risk status.

On an individual level, two women (10% of the LR group) demonstrated higher scores after screening. Both were low risk participants—one experienced bleeding following examination and the other received abnormal test results. The remainder showed no change or a reduction in anxiety levels.

### Pain

No significant differences in pain intensity experienced during EIS readings were observed between HRW and LRW. Average scores were low, with a mean VAS score of 0.97 for HR and 1.01 for LR participants (*p* = 0.94, Mann Whitney U), and a maximal score of 3.2 and 3.1 in each group respectively. When the ordinal PPI scores are considered, 90% of each group described either “no” or “mild” pain.

**Fig. 2:**
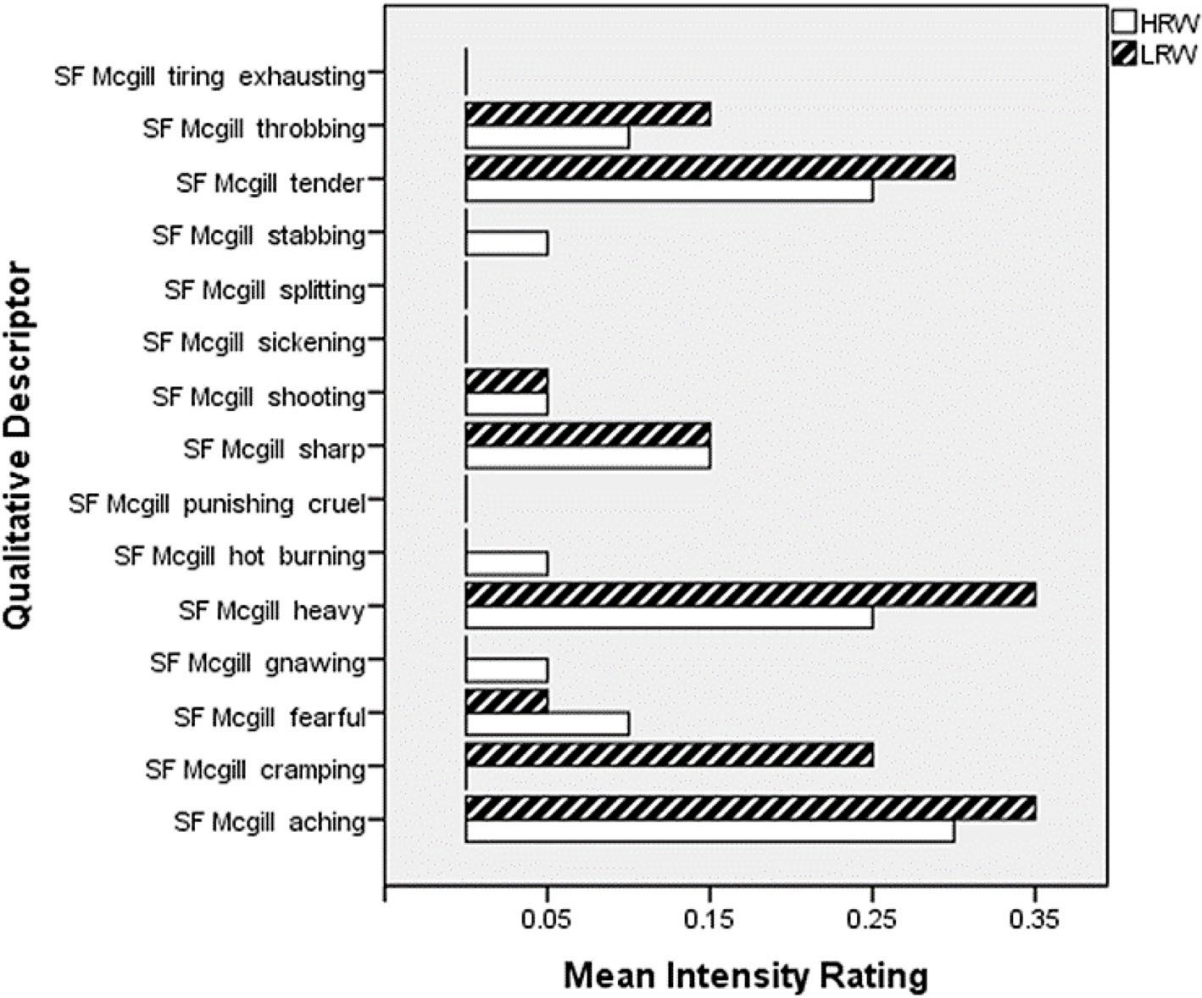
Differences in qualitative descriptor intensity rating (SF-McGill) between study groups

The results of the Pain Rating Index are summarised in Fig. 2, which displays the mean intensity rating for each qualitative descriptor in both sensory and affective domains, by study group. Women chose a broad range of descriptors, but the most commonly selected in both groups were “aching”, “heavy” and “tender”. However, it is notable that intensity ratings were almost exclusively 0 or 1 (no or mild pain), with only two scores of 2 (moderate pain) provided—one for the “tender” descriptor and the other for the “cramping” descriptor, by different low risk women. Affective descriptors were not commonly chosen by either group.

### Impression of EIS probe design

**Fig. 3:**
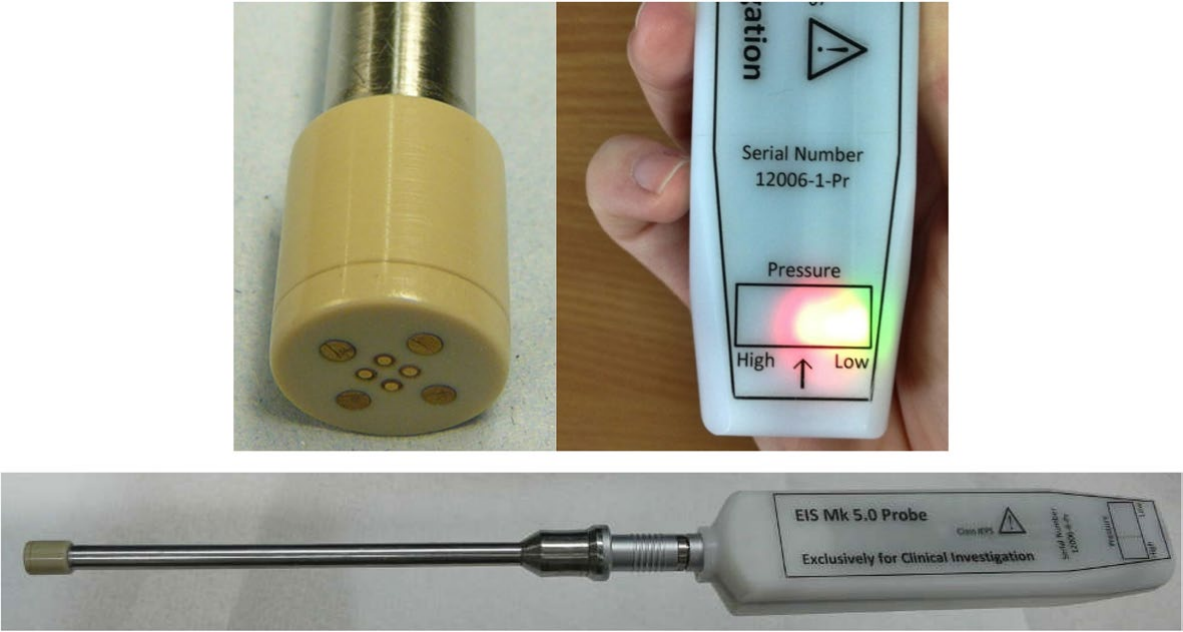
Appearance of EIS Probe (Sheffield Mark V)

75% of participants rated the appearance of the EIS device as 1 or less (VAS), with no significant difference observed between risk groups. Figure 3 illustrates the design of the Mark V probe used during clinical study visits.

### Overall personal acceptability rating and perspective on use in wider antenatal care

Having completed the anxiety, pain and device appearance ratings, participants were asked to appraise how acceptable they found the overall experience of undergoing EIS (via VAS) and whether they deemed it suitable for use antenatally. 100% of participants agreed EIS measurements would be acceptable for future use in antenatal care (binary rating). Mean VAS ratings were similar between groups (0.55 HR (range 0–3) *vs.* 0.75 LRW (0–5), *p* = 0.84, where 0 = acceptable, 5 = neutral and 10 = unacceptable).

## Qualitative analysis

The characteristics of the women who participated in the semi-structured interviews are summarised in Table 2. There was a preponderance of white British participants, although this was representative of the main study cohort. Effort was nevertheless made to capture the views of different ethnic groups, with support from a clinically experienced translator in one case. Women of different ages, socio-economic statuses and with varied obstetric histories were interviewed to capture as diverse a range of experience as possible. Participants ranged in age from 19 to 38 years and spanned the full range of indices of multiple deprivation (IMD) with 43% of participants residing in areas with IMD 1 to 5 and 57% with IMD 6 to 10. 76% were White British, 4.7% Black African, 4.7% Arabic, 4.7% South Asian, 4.7% White European and 4.7% White American.

**Table 2:** Characteristics of qualitative interview participants

Pt No	Location of interview	Age	Ethnicity	Marital staus	G	P	Gestation of previous preterm birth or miscarriage	Group	Having serial CL scans as well?	PTB prophylaxis?	Previous speculum exam?	Index of Multiple Deprivation (IMD) Decile^*^
1	Home	25	White European (Polish)	Co-habiting	2	1	n/a	Low risk	no	no	yes	5
2	University	33	White British	Married	5	0	Four 1^st^ trimester miscarriages	Low risk	yes	no	yes	10
3	University	21	White British	Co-habiting	2	1	n/a	Low risk	no	no	yes	7
4	University	36	White British	Married	3	2	Term birth then 31 week delivery	High risk	yes	no	yes	9
5	University	35	White British	Married	4	2	One 30 + delivery following term birth	High risk	yes	no	yes	6
6	University	19	White British	Co-habiting	4	2	One 36 + 0 delivery, term birth since	High risk	no	no	yes	7
7	University	38	White British	Married	2	0	One 1^st^ trimester miscarriage	Low risk	no	no	yes	5
8	University	28	Black African	Single	3	1	n/a	Low risk	no	no	yes	1
9	University	34	White British	Married	2	1	n/a	Low risk	yes	no	yes	8
10	University	29	White British	Married	1	0	n/a	Low risk	no	no	yes	4
11	University	33	White American	Married	3	2	One 29 + delivery, term birth since	High risk	yes	progesterone	yes	5
12	University	28	White British	Married	2	0	One 23 + delivery and neonatal death	High risk	yes	USS indicated suture	yes	9
13	Home	35	White British	Married	3	1	n/a	Low risk	no	no	yes	10
14	University	37	White British	Married	7	3	1^st^ trimester, 14/40 and 20/40 miscarriage. 2 term births before and once since miscarriages	High risk	yes	no	yes	1
15	University	30	White British	Married	3	2	One 35 + 5 delivery, term birth since	High risk	no	no	yes	10
16	University	29	Pakistani	Married	3	2	One 29 + one 27 week delivery	High risk	yes	progesterone	yes	1
17	Home	33	White British	Married	1	0	n/a	Low risk	no	no	yes	10
18	Antenatal ward	34	White British	Married	10	3	Recurrent 1^st^ trimester miscarriages + 23 week miscarriage + three 33–34 week deliveries	High risk	yes	USS indicated suture and progesterone	yes	7
19	University	37	Libyan	Married	6	3	One 21 week miscarriage + one 1^st^ trimester miscarriage, 3 term births since	High risk	no	elective cerclage	yes	3
20	Antenatal ward	36	White British	Co-habiting	8	3	30 and 32 week deliveries, term birth since	High risk	yes	progesterone	yes	2
21	University	30	White British	Married	2	0	n/a	Low risk	no	no	yes	9

Four over-arching themes were actively generated which summarised women’s accounts of undergoing EIS and the other screening tests: (i) the physical consequences of testing; (ii) emotional experiences during study visits and pregnancy; (iii) additional determinants of the screening experience and (iv) practical considerations regarding wider use of EIS. An overall synthesis of primary and secondary themes is provided in Table 3 with exemplar quotes to demonstrate each theme.

**Table 3:** Synthesis of interview themes

Main Theme	Sub-themes		Exemplar Quote
1) Physical consequences	1) Of EIS	Unable to feel measurements	*“Yes, I don’t- when she did the pen thing, I can’t remember what it’s called (I: The impedance?) Yes. I didn’t even feel anything, or notice anything that was happening. It was as if it hadn’t happened. But it had been done, because I didn’t even notice anything being done. So that was quite good.”* Participant 13 (HR, two MTLs, early miscarriage and three term births)
		Unusual sensation experienced	*“It’s like a bit of pressure I guess inside-it’s like nothing I have ever felt before. It’s kind of inside and up (laughs) but not painful just…just pressure, a strange kind of pressure which is not a normal feeling; you would not normally experience that.”* Participant 20 (LR, first pregnancy)
		Pain/discomfort/negative descriptors	*“…it sort of felt like I was getting an IUD *(intrauterine device)* put in. There’s a little pinch or a poke or something. But I think it’s the way, I think she moved it or something. So it wasn’t actually the instrument, it may have been the handling of the instrument.”* Participant 10 (HR, one PTB, one term birth)
		Positive descriptors	*“Couldn’t really feel much with that to be honest ummm I felt the swabs more and the speculum being placed than the impedance test, it was more like a very gentle pressure and then hearing the beeps so … yeah it wasn’t uncomfortable”* Participant 14 (HR, one PTB)
		Post-test symptoms	*“Nothing changed. I mean absolutely nothing changed. There was no bleeding, there was no discharge, I didn’t feel any aching. Nothing.”* Participant 18 (HR, one MTL and early miscarriage, three term births)
	2) Of other screening tests	Speculum	*“I think for me, the speculum is, not painful, but the most uncomfortable part of it.”* Participant 11 (HR, one 23 week delivery and neonatal death)
		Swabs	*“The swab is the one that’s, the swab is actually the one that’s uncomfortable… I mean you can feel it, you can sense it… you can actually feel it scraping, even though you know it’s a cotton swab and it’s just gentle”* Participant 18 (HR, one MTL and early miscarriage, three term births)
		TVUSS	*Yes, like I think the cervical scan is gentler than the rest of it, so I think it’s more having- and especially I think because I’ve had a lot of scans where they’ve used it to look at my ovaries, and they’ve been quite you know, whereas a cervical scan is much softer than that even, because they literally just want to gently go in and they can see everything. Whereas I’m used to cervical scans where you’re really looking for your ovaries and your follicles and stuff. So it’s really gentle. It’s almost like she only really needed to put a tiny bit of the tip in really, just to see what she needed to see* Participant 6 (LR, first pregnancy)
2) Emotional experiences	1) In relation to EIS	Uncertainty re: impending physical experience	*“It was definitely far less of a feeling or a pain feeling than I had expected. I expected to feel more invasive.”* Participant 2 (LR, recurrent first trimester miscarriages, first ongoing pregnancy)
		Concerns re: safety of novel test	*“I was a little bit, I have to say I was a little bit, you know because it’s research and someone’s checking, I sort of felt that if you’re taking part in something, you can’t completely say that there isn’t any risks. So that part of the research, I was anxious about that a little bit, but once I’d finished and sort of a couple of hours later, I wasn’t feeling any different, I mean it was fine* *…I wasn’t worried, but I was a little bit- It’s still a risk, it’s still, even though you’re guaranteed 99%, there’s always 1% of these going the opposite way.”* Participant 7 (LR, one term birth)
	2) In relation to other screening tests	Impact of visual result of CL scan	*“If she had just tried to explain that it is short, but seeing it myself on that screen, it’s made me realise that I can’t be messing about, I can’t be going home. I’ve got to listen to what they’re telling me to do.”* Participant 19 (HR, two PTB and one term birth)
		Psychological impact of CL scan results	*“It was just really reassuring to know exactly what was happening because you can’t feel anything can you with your cervix, so it’s impossible to know without the scans.”* Participant 11 (HR, one 23 week delivery and neonatal death)
		Psychological impact of FFN results	*“ The first study visit I did have a slight increase in fibronectin result…. which was a surprise and then a worry as well because obviously I didn’t expect anything to be picked up on it”* Participant 14 (HR, one PTB)
	3) During pregnancy in general	Fear and anxiety in pregnancy	*“Yes I think for me, it were like a blessing really, because I was already really paranoid about just being even pregnant. I think I was really, really scared”* Participant 9 (LR, first pregnancy)
		Falling through the gaps of antenatal care	*“P: You know if I’d not had all these tests done, I know for a fact I’d be thinking all the time, is that something? Is that something?* *I: What would you have done? Who would you have gone to?* *P: I don’t know. I probably would have just drove myself and my husband crazy I think (both laugh). I went to the doctors originally, and the mid-wife. And I explained to them about my anxiousness, and the fact that I didn’t know what had happened last time and how that was making me feel. And I felt that it got dismissed a little bit there.”* Participant 5 (HR, one term birth, one PTB)
	4) During high risk pregnancy	Emotional burden of previous obstetric trauma	*“I never actually think about it, because it’s been 5 years now. But you’re totally out of control. Like you can’t do anything. You can’t help your kid, you can’t do anything. You just have to like be there and it’s just not how life should begin, that stressful you know… I can’t even look at pictures of her, because she’s so tiny”* Participant 10 (HR, one PTB, one term birth)
		Cycle of anxiety in subsequent pregnancy	*“And then the day before I come in, apart from this time and last time, I had a really sleepless night because I’m thinking what is it going to show? What’s it going to show? And I can find myself just being laid wide awake, but then once I’d been I can sleep safe and sound again for a couple of weeks”* Participant 5 (HR, one term birth, one PTB)
3) Additional determinants of screening experience	1) The design of the EIS probe		*“P: I mean it’s sort of funny looking* *I: What do you mean by that?* *P: Well I think because it’s long and it’s like lights on it, and it makes a noise…* Participant 10 (HR, one PTB, one term birth)
	2) Perspectives on intimate examination	The vagina as a protected space	*“A speculum’s a bit uncomfy when you’re pregnant to kind of open you up a bit. And I suppose if you don’t have to have that done when you’re pregnant… Well you’d prefer not to have the speculum if you don’t have to”* Participant 6 (LR, first pregnancy)
		Intimate examinations as normal	“*I don’t feel it were, like there were no pain at all. It was literally just a bit uncomfortable. That’s all you can,* *well all I can really say about it, but other than that because it doesn’t hurt, because it’s just a normal thing.”* Participant 3 (LR, one term birth)
		Intimate examinations as beneficial	*“For me, it’s ok. It’s a little weird, but is not hurting, it’s not pain. I know that it’s just for good things. So I’m not worried…. Maybe that is uncomfy. But because it’s good reason to do it, because you need to know something, you just don’t mind.”* Participant 1 (LR, one term birth)
	3) Attitudes to knowledge in pregnancy	Pre-existing knowledge of preterm birth	*“so before I had my daughter, I didn’t even know you could deliver early”* Participant 10 (HR, one preterm birth, one term birth*)*
		“No-one knew why”	*“I’ve had a premature baby before, and the reasons for that birth were unexplained. So going into this pregnancy, I was quite anxious about it happening again and what may have caused it last time and things like that… you know if I’d not had all these tests done, I know for a fact I’d be thinking all the time, is that something? Is that something?”* Participant 5 (HR, one term birth, one PTB)
		“It’s good to know”	*“No I know, that’s what my sister says. She’s like ‘oh I don’t even want to think about it’. I’m like ‘yes do, like shit happens, you should know’… But see if I’m trying to think like before all this happened, if somebody offered me this, would I say yes? And I would, yes, I guess I would. Because you know, more knowledge is better than no knowledge.”* Participant 10 (HR, one PTB, one term birth)
	4) Screening environment		*“I had a blanket over my legs… and the door was locked, and she locked it so I could see she had locked it and there was a curtain and everything…”* Participant 20 (LR, first pregnancy)
	5) Interactions with clinical staff	Gender	*“She was talking, so she sort of made me feel comfortable, because we continued talking about something completely different to what we were doing. So I didn’t feel- I think the fact that she was a female made it slightly better too.”* Participant 7 (LR, one term birth)
		Explanation/ communication	*“She was very good at explaining all the way through what she was doing, what it was going to be like and things. It’s a little bit like the dentist we’ve got just now, he talks so that you know exactly what he’s doing, so you never are caught unaware like ‘what was that?’ or you know ‘that felt weird’ or whatever, because if you have that kind of dialogue through it then you know what you’re expecting and what’s going to happen”* Participant 12 (LR, one term birth)
		Bedside manner and rapport	*“I think as the weeks have progressed, I feel that we’ve got, I feel that we’ve built up quite a good rapport between us, and I do trust her. So she when she said things to me, I’ve been able to walk out and thing ‘well actually she’s told me it’s alright, so I’ve not got to worry about it until next time’. It’s very reassuring* Participant 5 (HR, one term birth, one PTB)
4) Practical considerations for broader implementation of EIS	1) Information leaflet		*“Like some of the bits I was like what is that? But most of it…. It was just all technical, well not technical but like, it were just like, I knew all the ins and outs of it so it weren’t too hard.….I would say to mum ‘what is that?’ ‘What’s that one mean?’ I can’t really remember all of it. I didn’t ignore it, I just read a bit of it.”* Participant 3 (LR, one term birth)
	2) Timing and frequency of screening		*“I think if it was at a time when you were coming to hospital anyway, like the 20 week scan, then I think that would be a really good idea. But like I was saying earlier, it kind of put me off taking part in the study before I had a premature labour, just because of work and commitments and thinking ‘oh I need to take more time off’…”* Participant 11 (HR, one 23 week delivery and neonatal death)
	3) Women’s opinions on overall acceptability for wider use in antenatal care	In favour of universal screening	*“…yes I think everybody should do it. I don’t know, sorry. I’m just a survivor of premature birth so I’m sort of for everything.”* Participant 10 (HR, one PTB, one term birth)
		Dependent on risk status	*“P: Whether I’d feel I would need to have it if I’ve gone through two, you know if I’ve had two kids already that haven’t had any premature-ness, then I don’t know if I’d feel the need if it was like you can have this or not have it* *I: If it was an option, you think that you would probably decline?* *P: Only in that I wouldn’t have the worry if myself to find out whether there was a risk of being premature. But I wouldn’t not have it if it was an offer I think, because the procedure wasn’t anything that you wouldn’t just say oh yes that’s fine, I can just have that as well so I know for sure that things are ok.”* Participant 12 (LR, one term birth)
		Trade-off between burden of tests and information gained	*“…you’d prefer not to have the speculum if you don’t have to. As a routine measure, it would be, but if it definitely picked up lots of, you know if it was going to pick up the risk of having a premature labour then yes it was definitely worth it, because it’s nothing compared to that”* Participant 6 (LR, first pregnancy)

### Physical consequences of testing

Women described the physical experience of screening in depth, with respect to both EIS and the other tests. The accounts of EIS were grouped into 5 sub-themes: “unusual” sensations; positive descriptions of measurements; pain/discomfort/negative descriptors; no sensation associated with measurement and post-test symptoms.

### Emotional experiences

Participants also detailed a range of emotions before and after study visits which inevitably shaped their overall perspective on their experiences. Some emotions related to EIS, with specific sub-themes of uncertainty regarding the impending physical experience and concerns regarding the safety of novel tests identified. However, others pertained to the conventional tests and specific sub-themes of general reassurance from screening; the visual impact of the CL result; and the specific psychological impact of CL scanning and FFN testing were evident.

### Additional determinants of screening experience

These included the design of the EIS probe, women’s pre-existing perspectives on intimate examination and attitudes to knowledge in pregnancy, the screening environment and interaction with clinical staff.

The sub-theme of perspectives on intimate examination incorporated two polarised stances: Some viewed the vagina as a protected space, with resultant caution regarding internal examination in pregnancy; others identified intimate examination as a normal, beneficial, part of pregnancy. Many felt that familiarity with intimate examination increases tolerance.

The ‘attitudes to knowledge in pregnancy’ subtheme draws together several concepts which all influence women’s perspectives on preterm screening, including prior knowledge and understanding of PTB, a lack of explanation for prior PTBs (where relevant) and the perception that knowledge during pregnancy is a good thing, in and of itself.

Women noted that provision of an appropriate, private screening environment positively impacted their experience. Similarly, clear communication and detailed explanation from the operator at all stages of screening was valued, as was continuity of care and the opportunity to build a rapport. Operator gender was also an important factor for several participants.

### Practical considerations regarding wider use of EIS

Some participants (in particular HRW) supported universal screening, whereas others preferred a risk based approach to offering additional tests. Several highlighted the need to balance costs and benefits of screening.

## Triangulation

### Anxiety

It is important to note that whilst assessments of pain and device design were relatively specific to EIS, anxiety ratings related more broadly to the screening package as a whole (and indeed to the pregnancy itself). Triangulation demonstrated general agreement between datasets regarding the reduction in anxiety after screening. However, it also detected context-specific examples of dissonance – notably in one woman who received a false positive fibronectin (level > 200 ng/ml though delivered at term) and another LR woman who felt that study participation had increased her awareness of (and therefore worry about) PTB. Both women qualified this by describing the net reassurance they obtained from participation, even though their anxiety was heightened at specific time-points. For example:

*“It just made me aware of that concern of premature birth that I haven’t really considered at all before. But now the tests are complete, I’m really glad I’ve taken part in them.”*

Participant 2 (LR, recurrent first trimester miscarriages, first ongoing pregnancy).

A variety of sub-themes were also identified during SSIs which help explain women’s anxiety and the emotional impact of the different screening tests.

As women and clinicians were blinded to spectroscopy results, there was no possibility of EIS itself providing direct reassurance about PTB risk. However, one EIS-related explanation for the observed trends was supported by complementary SSI data: namely, some women were anxious about the safety of undergoing a novel test in pregnancy but were reassured when they had no adverse experiences afterwards. This viewpoint was expressed by both HR and LRW. Uncertainty regarding the impending physical experience of EIS (or indeed other unfamiliar tests encountered during screening) similarly was a potential source of increased pre-test anxiety, as illustrated by participants 17 and 9:

*“the only thing I was concerned about or I’d thought about before was actually done was how much am I going to feel is it going to be like an electric shock kind of thing…”*

Participant 17 (HR, recurrent first trimester miscarriages, three PTBs, one MTL).

*“It wasn’t anything invasive or, you know, I think I expected it to be, you know the whole situation to be a bit uncomfortable, but it wasn’t*.”

Participant 9 (LR, first pregnancy).

Triangulation of individual items of the STAI-6 shows that many of the women’s emotional responses to testing were not specific to the EIS procedure (e.g. worry and anxiety related to abnormal tests, pre-test worries due to prior knowledge of PTB, e.g. due to family history). Moreover, the reassurance of receiving normal CL and FFN results contributed significantly to reduced post-test anxiety. When women did have worries resulting from positive test results they often framed this as a good thing (describing knowledge as good, and as a chance for action).

Additionally, the results of quantitative analysis suggested a possible bimodal distribution of pre and post-test STAI-6 scores amongst HRW (Supplementary Figs. 1a and 1b). Interview findings were concordant, with a subset of HRW providing detailed accounts of their marked pregnancy-related anxiety. Such emotions were not universally expressed, but when present, were a noticeable focus at interview. A range of complementary subthemes emerged, with HRW discussing reasons for their anxiety (lack of explanation of previous PTBs, the traumatic nature of previous pregnancies, difficulty accessing support from clinicians, fear of recurrent problems), their pattern of emotions (with cyclical anxiety in relation to appointments a strong theme) and their coping mechanisms (seeking information/explanation, developing trust and rapport through relationships with care givers, reframing abnormal test results as positive opportunities for action/preparation). HRW who had experienced later PTBs or positive outcomes following PTB did not typically express such strong emotions at interview. LRW were generally less emphatic in their expressions of anxiety and reassurance, consistent with questionnaire results. However, those who had undergone fertility treatment, experienced early miscarriage or with family history of PTB described higher anxiety. Nulliparity was also a source of worry for several LRW.

### Pain

Pain ratings also showed high concordance with interview data. Scores on both VAS and PPI scales were low, and similarly women made efforts to ensure their descriptions of the physical experience of EIS were not interpreted as pain during the SSIs. Phrases such as *“it’s not a pain at all”* (Participant 5), *“it’s not painful in any way”* (Participant 17) were often used as a prefix or suffix to more detailed descriptions. The qualitative descriptor most commonly used at interview was not included in the fifteen item McGill list: both HR and LRW frequently described a feeling of “*pressure*”. However, this may have been influenced by the real time explanations from the CRF during screening, as illustrated by Participant 3:

“*She said it would be a bit like pressure or something. I think she said pressure, something like that, but it weren’t, it were fine”*

Participant 3 (LR, one term birth).

The only discordant account noted at interview was that of one HRW (Participant 10), who described a higher degree of discomfort than anyone else (“*it felt like it poked, a sort of stabbing poke…*”, “*it sort of felt like I was getting an IUD put in*”). Interestingly her score on the VAS was 3 and on the PPI 2 (discomforting) which overall does not appear suggestive of high pain intensity (although her scores did represent the top of the range recorded for HRW). However, she too qualified her description (“*But I think it’s the way, I think she moved it or something. So it wasn’t actually the instrument, it may have been the handling of the instrument”),* perhaps suggesting a transient sensation at one reading, rather than a consistent sensation across all six readings at the two study visits*.*

Although items from the affective subscale of the McGill PRI were not commonly selected by questionnaire respondents, complementary themes from the interviews did emerge which detail the interplay between the emotional and physical experience of EIS. These include uncertainty regarding impending physical experience, concerns regarding the safety of a novel test and their perspectives on intimate examination. Women who expressed the opinion that checks and examinations were useful often found the physical experience particularly manageable—for example Participant 1, who recorded scores of 0 on both the VAS and the PPI:

*“For me, it’s ok. It’s a little weird, but is not hurting, it’s not pain. I know that it’s just for good things. So I’m not worried.”*

Participant 1 (LR, one term birth).

Similarly, some patients who had reflected upon the safety of EIS as a novel test recorded slightly higher pain scores, e.g. Participant 6 (VAS of 3 and PPI of 1) who stated:

*“I know it said that there wasn’t any harm with the impedance at all. But it would have been nice to have something in there that showed some evidence for that that backed it up like some statistics or previous pilot that says this has happened.”*

But also:

*“To be honest, I’m not sure, because the speculum was in, and I could feel the speculum, I can’t say that I massively felt anything. Maybe a little bit of like a tingle or like you were just doing a swab, just being touched kind of thing really”*

Participant 6 (LR, first pregnancy).

This slight conflict between pain score and qualitative account could imply that the emotional impact of EIS influenced women’s sensory experiences more than the questionnaire data suggests. Alternatively, despite the questionnaire aiming to establish the specific effects of EIS, her pain score may also reflect the discomfort experienced with speculum examination rather than the CR reading itself.

### Device design

Both methodologies yielded useful information concerning the design of the EIS device. The VAS scores spanned a wide range (from 0–9) although the majority (75%) of participants scored the probe appearance as non-threatening. The interviews confirmed this diversity of opinion; some women barely remarked upon the probe (indeed two said they couldn’t remember what it looked like, whilst another participant referred to “*the little pen thin*g”), whereas others expressed quite negative opinions regarding its appearance (using descriptors such as “*bulky”, “different”, “futuristic”, “odd, “intimidating”, “space age”, “scary”, “robot probe”).* The reflections of the latter group offer detailed insight into which features they found troublesome, including colour, length, the noise the probe made and its wireless connectivity.

### Test acceptability

Finally, the interviews provided significant complementary information regarding test acceptability. All women completing the questionnaire rated EIS as acceptable for use in antenatal care however, at interview, answers often related to the overall package of screening tests rather than being specific to EIS. Perceived acceptability was also clarified as being context specific by multiple participants. Some expressly advocated universal PTB screening (indeed, the highest risk women (with previous extreme or multiple PTBs/late miscarriages) provided the most emphatic support for this); others favoured application of tests to HR women only. These contrasting perspectives are illustrated by participants 17 and 2:

*“If they roll this out to people antenatally it would just become normal, as normal as having smear tests, it’s a really quick thing that could make such a difference.”*

Participant 17 *(*HR, recurrent first trimester miscarriages, three PTBs, one MTL).

*“I can imagine that if you weren’t having any intervention, it might feel quite, quite a serious thing to undertake..it might feel too much for the ordinary woman who wouldn’t expect to have medical intervention as part of a normal pregnancy”*

Participant 2 (LR, recurrent first trimester miscarriages, first ongoing pregnancy).

Many factors influenced these positions. Subthemes concerning knowledge in pregnancy and perspectives on intimate examination were particularly evident, as were women’s emotional experiences of both EIS and other screening tests. Participants who expressed concern about the safety of EIS and/or internal examination were more cautious about the idea of universal screening, in contrast to those who viewed both knowledge and intimate examination as beneficial who gained considerable reassurance from study tests. No women expressed reservations about the use of EIS in HRW – even those who had concerns about examination or EIS safety. HRW were frequently described as having most to gain from screening, which tipped the balance of test burden and benefit:

*“You have to think about the costs and the benefits don’t you?... I think for cases like mine where I have had a premature birth, then I think it would be very useful, if anything its reassurance for parents that things are being monitored”*

Participant 14 (HR, one PTB).

Thus overall, both datasets drew consistent conclusions regarding acceptability for use in HRW, whereas women’s qualitative accounts reveal some dissonance with respect to LR screening. Moreover, the qualitative accounts suggest that the quantitative acceptability ratings are influenced by the PTB screening package as a whole.

## Discussion

This study used a mixed-methods, parallel convergent approach to comprehensively evaluate women’s experiences of undergoing cervical EIS measurements as part of a PTB screening package. It is the first study to assess the acceptability of EIS and also contributes to the concise body of qualitative evidence regarding PTB screening in general. All participants deemed cervical EIS acceptable for use in antenatal care as part of a multimodal PTB screening package. It is encouraging to compare the binary rating of EIS acceptability with prior studies of PTB screening—these yielded similarly positive acceptability ratings for both CL scanning and FFN measurement by ≥ 90% of women [17, 18]. EIS was well tolerated (as evidenced by low pain scores and generally concordant SSI accounts). These findings compare favourably to existing data on pain experienced during CL screening (with mean VAS pain scores of 0.5 and 2.4 reported by Heath et al [16]. and Cicero et al [15]. respectively, and no or mild pain during TVUSS reported by the majority of participants in studies by Clement et al [17]. and Romero et al [19].). They provide useful information with which to counsel patients undergoing EIS measurements in future and may help address one of the potential sources of anxiety associated with screening, namely uncertainty about the impending physical experience of a novel test.

Interpretation of the data regarding anxiety is complex. Women’s emotional state varied according to risk status, prior experiences, the timing of assessment and their perspectives on intimate examination, novel tests and pregnancy in general. Overall, undergoing the whole package of screening was associated with a reduction in anxiety and many women with high anxiety levels were particularly emphatic in their appreciation of the reassurance gained through tests and monitoring. For HRW especially, comfort was not gained by the tests alone, but also through detailed explanations, regular attendance and the opportunity to build a rapport with care-givers. There is commonality between these SSI themes and preceding work: O’Brien et al*.*^*22*^ have previously noted the importance of the relationship between HRW and the PTB clinic team, and the role frequent checks play in breaking HR pregnancies down into manageable chunks. In both HR asymptomatic [22, 26] and symptomatic women [23, 24], prior studies have demonstrated comparably favourable views of increased surveillance and information provision in pregnancy to those expressed by our cohort.

The existing literature assessing anxiety during PTB screening is heterogeneous. Two studies have utilised the STAI-6 to evaluate the effect of CL scans [17] and FFN swabs [20] on maternal anxiety. However, they performed assessments at differing time-points and used single predictors in isolation, making direct comparison difficult. Nevertheless, the results from our cohort were broadly in keeping with those of Clement et al [17]., who noted significant reduction in worry about PTB after CL scanning. It is notable that women with a short CL were excluded from recruitment, thus they are likely to have over-estimated the reassurance provided by CL screening. In contrast, Shennan et al [20]. included women with both positive and negative test results in their assessment of FFN. Their finding of significantly increased anxiety in HR *vs.* LRW mirrors the trend reported in our cohort. Similarly, our isolated observation of increased anxiety post-screening in one LR participant with positive test results is in keeping with their observation that positive FFN swabs increase maternal anxiety. However, not every woman in our study with a short cervix or positive swab demonstrated an increased post-test STAI-6. It is plausible that undergoing more than one predictive test enabled women to reframe their results to mitigate against anxiety (e.g. by focusing on a normal CL if FFN was positive or, if both tests were abnormal, by reframing this knowledge as a positive opportunity for action).Therefore, detailed explanation is a vital element of PTB screening; Both risk groups in our cohort valued this, but it may not always occur in general clinical practice – Carlisle et al. noted a lack of understanding of PTB tests in symptomatic women, highlighting the need for clear communication when tests are used outwith a PTB clinic environment [25].

The experience of Participant 2 (the LR woman with heightened awareness of and worry about, PTB following recruitment) emphasizes the need for some caution when contemplating wider use of PTB screening. The advantages of awareness of PTB (increased knowledge of symptoms; empowerment to seek assessment and explanation; greater potential for mitigating/preparatory treatment) must be weighed against the potential disadvantages (e.g. provocation of intense or intrusive worry or anxiety which in itself might have physiological sequelae). The provision of contextual information (e.g. rates of all/very PTBs, management pathways for screen positive women) may help frame women’s risk perception, and explanation of how and where to seek help for concerning symptoms is essential. Overall, in this cohort no participant described marked adverse sequelae following screening, whereas HR women often gave vivid accounts of the shock and unpreparedness they felt during index PTBs. It is thus difficult to ascertain the net emotional impact of screening low risk women, but this certainly should be considered in the design and evaluation of potential screening programs. Although research thus far has failed to show benefit from screening low risk women, it remains possible that new predictive technologies may better identify those destined to deliver preterm or who may benefit from prophylaxis and that the evidence base for screening LRW may change over time. As a novel test, it was felt that EIS could offer improved accuracy of prediction, either as a standalone or additive test in both low and high risk groups. Screening approaches which only monitor women with high risk obstetric histories inevitably miss the opportunity to reduce the risk of an index preterm birth or late miscarriage, particularly in primiparous women. There is therefore a rationale to include low risk women in studies evaluating new predictive tests and for the reasons detailed above, assessing test acceptability to this group is important.

Although the design of the EIS probe was generally deemed satisfactory by study participants, the relatively wide range of scores yielded by the questionnaire suggested some diversity of opinion. The SSI data was therefore particularly useful with respect to this element of test acceptability and provided valuable insight into the specific aspects which some women found troublesome. Utilising this information to inform the design of future EIS devices will optimise the testing procedure for broader clinical use. Despite the consensus on EIS acceptability, women had varied opinions on routine screening. The theme of universal preterm birth screening was not introduced by the researchers, but was discussed by multiple participants at interview, particularly those who had experienced loss in previous pregnancies. This remains a controversial area, with disparity of international opinion. In the UK, the National Screening Committee advises against routine screening for asymptomatic low risk women [43]. However, in the United States, the American College of Obstetricians and Gynaecologists advises that all women undergo measurement of cervical length at the time of their anatomy scan^44^..No previous qualitative studies have examined women’s perspectives on who should be offered PTB screening, thus these findings provide an early insight into the views of a subset of both low and high-risk women. Further research is required to determine whether EIS might have a role in wider screening, either in isolation, or in combination with cervical length measurement.

This study is not without limitations. The sample size for the quantitative survey is modest and it is possible that more marked differences between risk groups would be observed within a larger cohort. In addition, the views reported here represent those who agreed to participate in the original clinical trial. Women declining recruitment could obviously not provide accounts of the screening tests but their views regarding EIS, PTB screening in general and their reasons for refusal may have provided useful information for future clinical policy and practice. Future work should aim to address this knowledge deficit by assessing the opinions of women who decline PTB screening. Whilst effort was made to capture the views of a diverse group of women, the subgroup who participated in the qualitative interviews was predominantly White British. Black and South Asian subgroups were relatively under-represented and, given their particular vulnerability to preterm birth, ongoing work should explicitly seek to clarify their perspectives on the acceptability of EIS. The exclusion of non-English speakers also limits the generalizability of our findings. Resource limitations impacted our ability to adapt study information, instruments and analytic techniques for application to a multilingual population, but we acknowledge the reporting gap this creates and would again to explicitly seek to address this in future work. Nevertheless, this is the first report on the acceptability of cervical EIS and, to our knowledge, the first study employing mixed methods to evaluate women’s experiences of PTB screening. Furthermore, prior qualitative work has focused on HR and symptomatic women – the inclusion of LRW in this study provides new insight regarding the tolerability and acceptance of PTB screening in a broader population of pregnant women.

## Conclusions

When used within a multi-modal screening package, EIS is an acceptable test to both high and low risk women. The physical experience of undergoing measurements was well tolerated by both groups. The emotional experience of testing was complex and influenced by many factors, some of which were unrelated to EIS measurement itself and stemmed from women’s previous pregnancy experiences, pre-existing attitudes to pelvic examinations and medical intervention, as well as their desire for information about their pregnancies. A theme common to many participants was that EIS acceptability was positively influenced by existing rapport between the operator and the woman. Gaining awareness of the way that these and other factors influence women’s experience of PTB screening will enable us to develop screening programmes which are acceptable to as many women as possible. This in turn will maximise the effectiveness of any future screening programme.

## Supplementary Information

**Additional file 1.** 

**Additional file 2.** 

**Additional file 3.** 

**Additional file 4.**

## Data Availability

The dataset used for the quantitative analysis of the acceptability questionnaires is available in additional file 4. Patient consent to share full transcripts of the semi-structured interviews was not obtained during recruitment and complete anonymisation is not feasible as the transcripts include discussion, for example, of prior obstetric history. Therefore, this dataset is not publicly shared but further detail is available from the corresponding author on reasonable request.

## Abbreviations

EIS: Electrical impedance spectroscopy
STAI-6: Six question short form of Spielberger State Trait Anxiety Inventory
PTB: Preterm birth
TVUSS: Transvaginal ultrasound scan
CL: Cervical length
FFN: Fetal fibronectin
QUiPP: Quantitative Instrument for the Prediction of Preterm Birth
LRW: Low-risk women
HRW: High-risk women
VAS: Visual analogue scale
PPI: Present pain intensity
PRI: Pain rating index
TA: Thematic analysis
