# Supplementary material for: The acceptability of cervical electrical impedance spectroscopy within a multi-modal preterm birth screening package: a mixed methods study
Source: BMC Pregnancy Childbirth. 2022 Dec 22;22:959. doi: 10.1186/s12884-022-05202-z (PMC9783720; doi:10.1186/s12884-022-05202-z)
Supplement: Supplementary file 1 — Additional file 1. [file 12884_2022_5202_MOESM1_ESM.docx]

**Additional file 1 – Semi-structured interview schedule**

1. Were there any reasons why you wanted to take part in the study?
2. Had you had any similar checks or examinations before?
3. What were your thoughts about premature birth before the study was discussed with you?
4. How did you find the research visit?
5. Can you remember what information you were given before and during the visit? How did you find the information?
6. Can you remember the different bits of the tests?
7. How did you find the different tests?
8. What were your experiences after the tests?
9. Can you remember what you were told about your test results?
   - 1. How did you feel about them?
     2. If negative effects: what did you do?
10. What would you think about having the tests again?
11. Is there anything else you want to discuss/let us know about?
