# Supplementary material for: The acceptability of cervical electrical impedance spectroscopy within a multi-modal preterm birth screening package: a mixed methods study
Source: BMC Pregnancy Childbirth. 2022 Dec 22;22:959. doi: 10.1186/s12884-022-05202-z (PMC9783720; doi:10.1186/s12884-022-05202-z)
Supplement: Supplementary file 2 — Additional file 2. [file 12884_2022_5202_MOESM2_ESM.docx]

Convergence Coding Matrix for Mixed Methods Analysis of Acceptability Data:

| **Quantitative Domains** | | | | | | **Triangulation Results (comparing QUANT with QUAL)** | **Qualitative Themes**  **(help explain the why)** | **Sample Quotes** |
| --- | --- | --- | --- | --- | --- | --- | --- | --- |
| **Spielberger State Trait Anxiety Inventory** | | | | | | | | |
| - Both HR and LRW showed significantly lower STAI scores after vs. before testing. - HRW demonstrated higher pre-visit STAI scores but also a larger mean reduction in STAI score than LRW - HRW were significantly calmer, less tense, more relaxed and less worried following screening. - LRW were significantly less tense and more content following testing | | | | | | | | |
| **STAI-6 anxiety**  **Pre & post test** |  | | I feel calm  Pre-test HRW | | | Complementary | Interactions with clinical staff – explanation and bedside manner | *“I was put at ease to begin with, and explained to me what would happen and everything like that.”* (Participant 13 HR, two MTLs, early miscarriage and three term births) |
|  |  | | I feel calm  Pre-test LRW | | | Complementary | Intimate examinations as beneficial | *“I know that it’s just for good things. So I’m not worried.”* (Participant 1, LR, one term birth) |
|  |  | | I feel calm  Post-test HRW | | | Complementary | Cycle of anxiety in subsequent pregnancy  Emotional burden of previous obstetric trauma  Reassurance of other screening tests | *“…but then once I’d been I can sleep safe and sound again for a couple of weeks”* (Participant 5, HR, one term birth, one PTB)  *“Yes, a lot calmer. I got more panicky before 20 weeks than I was normally. I was a lot more calmer, I wasn’t as worried about things. So it helped out a lot with that.”* (Participant 13 HR, two MTLs, early miscarriage and three term births) |
|  |  | | I feel calm  Post-test LRW | | | Agreement | Reassurance of other screening tests | *“But having now completed that study, it’s nice that that’s almost something to be ticked off, not in their area of concern and has helped me feel a lot more calm and content about the pregnancy”* (Participant 2, LR, recurrent first trimester miscarriages, first ongoing pregnancy) |
|  |  | |  | | | Complementary | Fear and anxiety in pregnancy  Reassurance of other screening tests | *“I had a personal experience. I had a bit of a fear, because my sister has got a problem with her cervix…*  *“…But I was really, you know, really reassured me that actually I would be alright, you know, I’d not got similar symptoms as my sister”* (Participant 9, LR, first pregnancy) |
|  |  | | I am tense  Pre- test HRW | | | Complementary | Cycle of anxiety in subsequent pregnancy | *“And then the day before I come in, apart from this time and last time, I had a really sleepless night because I’m thinking what is it going to show? What’s it going to show? And I can find myself just being laid wide awake”* (Participant 5, HR, one term birth, one PTB) |
|  |  | | I am tense  Pre test LRW | | | Complementary | Concerns re: safety of novel test | *“I was a little bit tense, I have to say I was a little bit, you know because it’s research and someone’s checking, I sort of felt that if you’re taking part in something, you can’t completely say that there isn’t any risks. So that part of the research, I was anxious about that a little bit, but once I’d finished and sort of a couple of hours later, I wasn’t feeling any different, I mean it was fine”* (Participant 7, LR, one term birth) |
|  |  | | I am tense  Post test HRW | | | Silence | - | *-* |
|  |  | | I am tense  Post test LRW | | | Silence | - | *-* |
|  |  | | I feel upset  Pre test HRW | | | Silence | - | *-* |
|  |  | | I feel upset  Pre test LRW | | | Silence | - | *-* |
|  |  | | I feel upset  Post test HRW | | | Complementary | Psychological impact of FFN results | *“I was a bit upset about it because as I say I wasn’t expecting that to pick up abnormalities (positive FFN) but it was nothing that wasn’t kind of dealt with…”* (Participant 14, HR, one PTB) |
|  |  | |  | | | Disagreement | Interactions with clinical staff – explanation  Reassurance of other screening tests | *“I have never gone away thinking I have no idea what went on or I am really upset about this there has never been any upset or distress whenever I have left I have always felt better than I did when I came”* (Participant 17*,* HR, recurrent first trimester miscarriages, three PTBs, one MTL) |
|  |  | | I feel upset  Post test LRW | | | Silence | - | *-* |
|  |  | | I am relaxed  Pre test HRW | | | Complementary | Interactions with clinical staff – Bedside manner and rapport | *“(*The CRF*) has such a lovely bedside manner that she just makes me feel really relaxed”* (Participant 11, HR, one 23 week delivery and neonatal death) |
|  |  | | I am relaxed  Pre test LRW | | | Agreement | Intimate examinations as normal | *“I knew I was having these tests and everything. I was quite relaxed about it anyway in advance; I wasn’t worried about it at all…”* (Participant 20*,* LR, first pregnancy) |
|  |  | |  | | | Complementary | Interactions with clinical staff – Bedside manner and rapport | *“she made me feel really comfortable and relaxed”* (Participant 20*,* LR, first pregnancy) |
|  |  | | I am relaxed Post test HRW | | | Complementary | Reassurance of other screening tests | *“But then, once obviously you get that result (negative FFN) that’s quite reassuring, and that’s put my mind at ease for the next two weeks and I become really relaxed.”* (Participant 5, HR, one term birth, one PTB) |
|  |  | |  | | | Agreement | Reassurance of other screening tests | *“everything’s fine, it’s positive and she’s relaxed”* (Patient 15, HR, two PTBs) |
|  |  | |  | | | Complementary | Attitudes to knowledge in pregnancy - “It’s good to know” | *“So that put me at ease as well, knowing that everything had been checked, and nothing was going wrong that they could tell, so that was good”* (Participant 13, HR, two MTLs, early miscarriage and three term births) |
|  |  | | I am relaxed  Post test LRW | | | Complementary | Uncertainty re: impending physical experience | *“I think I expected it to be, you know the whole situation to be a bit uncomfortable, but it wasn’t. It was, yes just quite at ease”* (Participant 9, LR, first pregnancy) |
|  |  | | I feel content  Pre test HRW | | | Silence | - | *-* |
|  |  | | I feel content  Pre test LRW | | | Silence | - | *-* |
|  |  | | I feel content  Post test HRW | | | Agreement | Reassurance of other screening tests | *“I: And then when you get the result, how do you feel?*  *P: Happy and relaxed”* (Patient 15, HR, two PTBs) |
|  |  | |  | | | Agreement | Interactions with clinical staff – explanation | *“I have never gone away thinking I have no idea what went on or I am really upset about this there has never been any upset or distress whenever I have left I have always felt better than I did when I came”* (Participant 17*,* HR, recurrent first trimester miscarriages, three PTBs, one MTL) |
|  |  | |  | | | Complementary | Attitudes to knowledge in pregnancy - “It’s good to know” | *“I remember when she told me about the results I was actually quite pleased, I was like ‘oh this is actually nice’, you know you feel like you’re getting extra care, I mean it*  *was nice. I wasn’t expecting it before, but it was really nice later on when she told me that what they were measuring was actually a predictor and everything. And especially when you’re sort of considered higher risk, it’s actually nice to know I suppose to have that reassurance.”* (Participant 18, HR, one MTL and early miscarriage, three term births) |
|  |  | | I feel content  Post test LRW | | | Complementary | Impact of visual result of cervical length scan  Interactions with clinical staff – explanation and bedside manner | *“I thought that were nice. I thought it was lovely to see the baby’s feet and I were looking at it from a different point of view, I suppose, and I suppose you see a part of the body that you don’t normally see. And she was explaining about the cervix, and I think that the way she did it was really good…*  *“I thought it was really good, I’m pleased I got the chance to take part.”* (Participant 9, LR, first pregnancy) |
|  |  | |  | | | Complementary | Reassurance of other screening tests  Attitudes to knowledge in pregnancy - “It’s good to know” | *“But having now completed that study, it’s nice that that’s almost something to be ticked off, not in their area of concern and has helped me feel a lot more calm and content about the pregnancy”* (Participant 2, LR, recurrent first trimester miscarriages, first ongoing pregnancy) |
|  |  | |  | | | Complementary | Psychological impact of cervical length scan | *“I: Ok, so how did you find getting the results…?*  *P: No, when she said that on the screen everything is pretty good. So I was fine, I was feeling good about that. I’m not like waiting for the result, but when I see it, that was also good that everything is ok”.* (Participant 1, LR, one term birth) |
|  |  | | I am worried  Pre test HRW | | | Complementary | Emotional burden of previous obstetric trauma  Attitudes to knowledge in pregnancy - “No one knew why”  The vagina as a protected space  Uncertainty re: impending physical experience  Cycle of anxiety in subsequent pregnancy | *“There is just anxiety related to the premature delivery and those kinds of things but, it’s more... I guess it was more the worry, but for us there wasn’t much explanation about why what had been happening was happening…*  *“I did have a little bit of reservations about having it done, with it being internal at first. I thought what will that be like? And will it alter things..? I just think it’s just the thought of being poked and prodded and if that would activate the baby, and if that might make the baby come on straight away…*  *“It was like just a bit of anxiety really about what you know, what might go wrong…*  *“I think that’s because I’m quite an anxious person really when it comes to, well I felt very anxious at first, so I just wanted to make sure that I was doing the right thing really.”*  *“When you’re waiting for the results, you feel really anxious. But, especially the first couple of times because every time I’d been coming in, it had been, I think I’d been on the shorter cervix scale prior…”* (All quotes Participant 17*,* HR, recurrent first trimester miscarriages, three PTBs, one MTL) |
|  |  | |  | | | Agreement | Cycle of anxiety in subsequent pregnancy | *“About 2 days before, she started getting anxieties and more anxious”* (Patient 15, HR, two PTBs) |
|  |  | | I am worried  Pre test LRW | | | Complementary | Pre-existing knowledge of preterm birth | *“In a way it has created a wee bit of worry about that particular issue because it’s not of premature birth, because it’s not one that I had considered a concern from my perspective before and my history.”* (Participant 2, LR, recurrent first trimester miscarriages, first ongoing pregnancy) |
|  |  | |  | | | Agreement | Pre-existing knowledge of preterm birth | *“I had a bit of a fear, because my sister has got a problem with her cervix, so she had twins early”* (Participant 9, LR, first pregnancy) |
|  |  | |  | | | Complementary | Intimate examination as beneficial | *“I know that it’s just for good things. So I’m not worried.”* (Participant 1, LR, one term birth) |
|  |  | |  | | | Disagreement | Concerns re: safety of novel test | *“I wasn’t worried, but I was a little bit- It’s still a risk, it’s still, even though you’re guaranteed 99%, there’s always 1% of these going the opposite way”* (Participant 7, LR, one term birth) |
|  |  | | I am worried  Post test HRW | | | Complementary | Psychological impact of CL scan | *“She’s a bit worried today, only because the result (CL scan) was slightly changed, but she’s otherwise, you know, she’s fine”* (Patient 15, HR, two PTBs) |
|  |  | |  | | | Complementary | Psychological impact of FFN results  Attitudes to knowledge in pregnancy - “It’s good to know” | *“The first study visit I did have a slight increase in fibronectin result…* *which was a surprise and then a worry as well because obviously I didn’t expect anything to be picked up on it.…*  *“…obviously we found out that our result is this but had we had not done the study we would not have known anything about it so I wouldn’t have that to think about and worry about, but….. I did know about it, but that was a good thing really because it was getting checked out and if we were starting to find that there were issues then I was being seen by people who could sort things out…”* (Participant 14, HR, one PTB) |
|  |  | |  | | | Complementary | Attitudes to knowledge in pregnancy - “It’s good to know”  Psychological impact of CL scan | *“so I still feel worried that something’s going to happen, that it’s going to come early. But it’s sort of reassured me, knowing what’s going off down there* (a short cervix) *do you know what I mean?”* (Participant 19, HR, two PTB and one term birth) |
|  |  | | I am worried  Post test LRW | | | Complementary | The vagina as a protected space  Concerns re: safety of novel test  Design of the EIS probe | *“But I just thought well whenever you have a vaginal examination you feel a bit uncomfortable afterwards, so had I not been pregnant, no it wouldn’t have worried me at all. But obviously being pregnant, you get a lot more worried, because you don’t want it to affect anything in your pregnancy. But that might be because I’m a bit anxious about that”* (Participant 6, LR, first pregnancy)  *“I: But you didn’t like the thought of it being connected?*  *P: Maybe, but only because I wasn’t aware of it. I didn’t realise it would work like that, yes. Only because there’s all these things on mobile phones, you don’t know a lot of stuff do you about it.*  *I: So you’re worried about the effects and the possibility that it could have an effect that we don’t know about?*  *P: Well that sounds a bit bad, I suppose, but yes, maybe. But I’m sure it’s fine”.* (Participant 6, LR, first pregnancy) |
|  |  | |  | | | Complementary | Interactions with clinical staff – communication  Fear and anxiety in pregnancy in general | *“She just rang me and had a chat, and said there was nothing to worry about obviously. It was just, ‘you’ve got a bit of thrush’. She didn’t leave a voicemail or anything like that so that was good because I have had them in life where they’ve done that with different things though. And it is, you do panic. So especially a first time mum, I think you do panic. Anything anyone says to you you’re a bit like *panic sound*, whereas actually, she did just, it wasn’t a bit issue obviously, but it was good the way that she rang to tell me.”* (Participant 9, LR, first pregnancy) |
| **Short Form McGill Pain Questionnaire** | | | | | | | | |
| - Average scores were low, with a mean VAS score of 0.97 for HR and 1.01 for LR participants (p=0.94, Mann Whitney U), and a maximal score of 3.2 and 3.1 in each group respectively. - When the ordinal PPI scores are considered, two women in each group (10%) rated their pain intensity during EIS measurement as “discomforting”. The remainder described either “no” or “mild” pain (35 and 55% of HRW and 45 and 45% of LRW - Women chose a broad range of descriptors, but the most commonly selected in both groups were “aching”, “heavy” and “tender”. Intensity ratings were almost exclusively 0 or 1 (no or mild pain), with only two scores of 2 (moderate pain) provided - one for the “tender” descriptor and the other for the “cramping” descriptor, by different low risk women. | | | | | | | | |
| **VAS and PPI (continuous and nominal ratings of pain intensity)** |  | | HRW | | | Agreement | Unable to feel the measurements  Positive descriptions of measurements | *“I didn’t feel it at all”* (Participant 4, HR, one term birth, one PTB)  *“It’s not painful”.* (Patient 15, HR, two PTBs)  *“it’s not painful in anyway, you are kind of aware its being done...and again mine was…. just because it was me (laughs) the wire was playing up so she had to do it twice to get a reading umm…but that actual bit of it isn’t uncomfortable really.”* (Participant 17*,* HR, recurrent first trimester miscarriages, three PTBs, one MTL) |
|  |  | |  | | | Complementary | Unusual sensation | *“not anything that was really awful, just I could feel something”* (Participant 11, HR, one 23 week delivery and neonatal death)  *“The most uncomfortable one is probably the newest one...*  *it’s not a pain at all. It is literally just a sensation of pressure just for a few seconds, then you know, it’s finished”* (Participant 5, HR, one term birth, one PTB) |
|  |  | |  | | | Disagreement | Pain/discomfort/negative descriptors | *“it sort of felt like I was getting an ID put in. There’s a little pinch or a poke or something. But I think it’s the way, I think she moved it or something. So it wasn’t actually the instrument, it may have been the handling of the instrument. But I said ‘Ah!’ and I have a pretty high tolerance for pain”* (Participant 10, HR, one PTB, one term birth) |
| **VAS and PPI (continuous and nominal ratings of pain intensity)** |  | | LRW | | | Agreement | Unable to feel the measurements  Positive descriptions of measurements | *“it were fine, it didn’t hurt. I couldn’t really tell any difference”* (Participant 3, LR, one term birth)  *“To be honest, I’m not sure, because the speculum was in, and I could feel the speculum, I can’t say that I massively felt anything…. So it wasn’t really uncomfortable I don’t think, from what I can remember”* (Participant 6, LR, first pregnancy)  *“I didn’t feel anything.”* (Participant 7, LR, one term birth)  *“it didn’t hurt or anything”* (Patient 16, LR, one term birth) |
|  |  | |  | | | Complementary | Uncertainty re: impending physical experience  Unusual sensation | *“There was just a little bit of discomfort, but it was so minor… it was definitely far less of a feeling or a pain feeling than I had expected. I expected to feel more invasive. But not, it was fine from my perspective, and again so short lived that they didn’t feel like I needed it to stop”* (Participant 2, LR, recurrent first trimester miscarriages, first ongoing pregnancy)  *“Just a little bit uncomfortable, a bit weird”* (Participant 20*,* LR, first pregnancy) |
| **Pain rating index** |  | | HRW | | | Complementary | Unusual sensation  Pain/discomfort/negative descriptors  Positive descriptions of measurements | *“it’s like you can just feel a little bit of pressure, because it pulsates. But it’s literally for a minor, it’s just a matter of seconds…*  *you can just feel that pulsation a little bit up, it just feels like it’s up in your tummy. That’s what it feels like (I: Does it?) Yes. But I wouldn’t say that it’s discomfort, it’s more of a pressure. That’s what it feels like, but it’s light pressure.*  *But it’s just the fact that you know it’s different to the other things. It’s a different sensation”.* (Participant 5, HR, one term birth, one PTB)  *“It just felt like it poked, like a stabbing poke”.* (Participant 10, HR, one PTB, one term birth)  *“Well I was looking at the ratings of the pain, you know on the questionnaire? And I think that’s the only one I could describe it as similar. Just something that is not a normal feeling, but just felt a bit like burning. But just for a split second and not really painful burning. Sorry it’s probably a bad description.*  *it was more like a very gently pressure and then hearing the beeps so ... yeah it wasn’t uncomfortable”* (Participant 11, HR, one 23 week delivery and neonatal death)  *“No pressure, just funny”.* (Patient 15, HR, two PTBs)  *“You can just feel a little bit of pressure and very minimally, it’s not painful in anyway, you are kind of aware its being done”* (Participant 17*,* HR, recurrent first trimester miscarriages, three PTBs, one MTL) |
| **Pain rating index** |  | | LRW | | | Complementary | Unusual sensation | *“Pressure, throbbing maybe, bit like, it’s just like how can you describe it? You know when you pinch? Like that, just kind of like that. But not hard. It’s just like a literally, like that. Nothing more than that I’d say”*  *“It’s like a bit of pressure I guess inside-it’s like nothing I have ever felt before. It’s kind of inside and up (laughs) but not painful just…just pressure, a strange kind of pressure which is not a normal feeling; you would not normally experience that”* (Participant 20*,* LR, first pregnancy) |
| **Design of device** | | | |  |  |  |  |  |
| Most participants (75%) rated the appearance of the EIS device as 1 or less (on a ten-point visual analogue scale where 0 = not threatening, 5 = neutral and 10 = very threatening). However, there was a wide range of scores, from 0 to 9. Average scores for HR and LRW were not significantly different: mean rating 1.3 for HRW (range 0-5) and 1.35 for LR (range 0-9) (p=0.98). The two participants who rated the EIS device as most threatening (with scores of 6 and 9) were low risk women. | | | | | | | | |
| **VAS rating** |  | | HRW | | | Complementary | Other determinants of screening experience – the design of the EIS probe | *“That’s the little pen thing?...*  *You can hear the bleep for when it finishes, but half the time I didn’t even know it was that, because it bleeps at the end.”* (Participant 13, HR, two MTLs, early miscarriage and three term births)  *“P: Just a bit space age, but it’s no worse than the scan thing. It’s quite intimidating. (I: Did you find the scan thing more intimidating?) P: Yes.“* (Participant 4, HR, one term birth, one PTB)  *“But then you have this like, it looks like a sword that makes you know, noise…*  *Yes it could be visually more appealing. Like I, so every two weeks I have the ultrasound, that doesn’t look scary, it just looks like a big plastic knob. But this is, it looks very futuristic.”* (Participant 10, HR, one PTB, one term birth) |
| **VAS rating** |  | | LRW | | | Complementary | Concerns re: safety of novel test  Perspectives on intimate examination – Intimate examinations as normal | *“the last test does feel quite alien almost compared to other tests which were just standard smear tests or a standard scan… Yes I think it’s because of the equipment more than anything. Most people are used to having swabs, used to yes, having the equipment of a transvaginal check, but it just feels like something odd to be near you *laughs*.”*  *“Yes, a buzz and then a, was it a beep? Yes, I suppose the noises are very harsh noise”* (Participant 2, LR, recurrent first trimester miscarriages, first ongoing pregnancy)  *“I noticed that it connected to the internet, and didn’t really realise that until after, which felt a bit strange having something connected to the internet whilst inside you, if that makes sense. I don’t know, that was a bit weird. But just so close to your baby and everything, you know, you don’t really want lots of frequencies and things like that, I suppose”* (Participant 6, LR, first pregnancy)  *“I mean it looks a bit scary. It looks a bit like a robot probe thing, so not very, yes it looks really medical… I don’t know, maybe it should be white or something. You know like maybe match the scan machines maybe. Because you know the scan thing, that it doesn’t look particularly scary. You can see it’s medical equipment but it doesn’t look scary.”* (Participant 8, LR, one term birth, previous colposcopy) |
| **Overall Acceptability rating and assessment of acceptability for use in AN care** | | | | | | | | |
| - For the VAS rating of acceptability there were no significant differences between study groups: mean rating for HRW 0.55 (range 0-3) and LRW 0.75 (range 0-5) (p=0.84). - On the questionnaire only two participants provided additional comments in the free text section regarding acceptability: one mentioned that her procedure was slightly prolonged due to issues with device connectivity, but did not find this unacceptable, the other stated that she wouldn’t wish to undergo the test routinely but would be willing to do so if clinically indicated. - 39/40 women indicated they felt no change to the EIS procedure was necessary, whilst one would have preferred it if the test could be performed without using a speculum - When asked if the procedure would be acceptable for use in antenatal care, there was universal agreement from all surveyed women, although the question did not specify in what context this use might occur (e.g. high risk vs universal screening). | | | | | | | | |
| **Binary rating and VAS** | |  | HRW | | | Agreement | In favour of universal screening  Attitudes to knowledge in pregnancy - “It’s good to know” | *“But yes I think it’s a really good idea to offer it.”* (Participant 11, HR, one 23 week delivery and neonatal death)  *“It’s definitely...if this was available to people, or even for additional people for the study at least go and find out about it and make you r own decision and go and meet the team and be explained to and have those first preliminary checks done and things and see how they feel about it then before rolling it our completely. As somebody that potentially… if they roll this out to people antenatally it would just become normal, as normal as having smear tests, it’s a really quick thing that could make such a difference.”* (Participant 17*,* HR, recurrent first trimester miscarriages, three PTBs, one MTL)  *“As somebody who’s had a premature birth, I would do anything and everything, so yes I would do it. But if I didn’t have a premature birth, I would want to know does it actually help determine if I may go into labour? You know what I’m saying, because like it’s not that it’s invasive, but if you, I could see how other people would not want to do it. Because I know a lot of my friends are like ‘I don’t want anything going on in there, I don’t even want to have sex because you know that’s where the baby’s going to come out’. But I don’t know, yes I think everybody should do it. I don’t know, sorry. I’m just a survivor premature birth so I’m sort of for everything. But see if I’m trying to think like before all this happened, if somebody offered me this, would I say yes? And I would, yes, I guess I would. Because you know, more knowledge is better than no knowledge. So then yes. Yes and yes.”* (Participant 10, HR, one PTB, one term birth) |
|  | |  | HRW | | | Complementary | Yes for high risk women  Trade off - burden of tests (physical, practical) & information gained | *“I think for cases like mine where I have had a premature birth, then I think it would be very useful, if anything its reassurance for parents that things are being monitored”* (Participant 14, HR, one PTB)  *“only the people that have experienced a premature”* (Patient 15, HR, two PTBs)  *“I think if people, because obviously I was in an at risk category from having colposcopy, and I think that if that had been offered to me the first time, rather than having all of the scans, probably I would have gone for that first, rather than the scans, because the scans are very invasive every two weeks, aren’t they? That’s what I have, every two weeks”* (Participant 8, LR, one term birth, previous colposcopy)  *“you have to think about the costs and the benefits don’t you?”* (Participant 14, HR, one PTB) |
|  | |  | HRW | | | Disagreement | LR women might be less accepting of screening | *“I: Do you think it would be beneficial you know for women who have never had a premature delivery, what do you think about those women?*  *P: No I don’t. My first pregnancy, it was normal, and I never, the thoughts of anything like this happening never entered my head.*  *I: So if somebody offered you the test then, what do you think you would have said?*  *P: I don’t know. I think I would have probably said no I’m alright if I’m honest.”* (Participant 4, HR, one term birth, one PTB)  *“I think there would probably be a bit more reservation about it, because just from speaking to my friends when I was considering it originally, they were like ‘Ooh I don’t think you should have it done, I think you should just leave things be’. So I think for a lot of women, the thought of being poked and prodded doesn’t really appeal to them.”*  (Participant 5, HR, one term birth, one PTB)  *“if it was offered routinely I don’t know how many people would want to do it because it is a more invasive test, if that makes sense?”* (Participant 14, HR, one PTB) |
| **Binary rating and VAS** |  | | LRW | | | Agreement | In favour of universal screening  Attitudes to knowledge in pregnancy - “It’s good to know” | *“Yes it can be because it’s like nothing more to- it’s just a scan and swab, just 15 minutes and it can be like a huge step in a, not education but you will know about your body more than you can know. Because if for example, me, if there’s something wrong there, on normal scan you don’t have to see that, so that’s better for all women.”* (Participant 1, LR, one term birth)  *“Because obviously you know don’t you? Like I was going to do this anyway because obviously it just helps anyway, but like if you think about it, you find out more. It’s more to your benefit if anything because if there is anything wrong, you know then don’t you? You can like, you can always get sorted out. So I think it’s a right good idea.”* (Participant 3, LR, one term birth)  *“I: So do you think it could be brought in as part of the routine care?*  *P: I believe they should, yes.*  *I: Do you think if it had been your first pregnancy you would have taken part?*  *P: Yes, yes. But I don’t know if that’s because of my background. I think my background (*nursing) *also takes a part to my decision making, so I would have yes.”* (Participant 7, LR, one term birth)  *“I: So you think it could be used in routine pregnancy?*  *P: Yes I do, I think it’s a good- I think as well with anybody, if there’s any nasty’s up there or any bugs or anything, I think it’s good to treat things. I don’t think it’s good to leave undone. And also it’s really informative, it was really informative I felt, and I felt really reassured after I’d had the test, yes. I felt really reassured that it’s going to be ok because it was obviously people think it’s about getting to 12 weeks, but nobody really hears about the 20 weeks or what brings that on, so there’s another side isn’t there?”* (Participant 9, LR, first pregnancy) |
|  |  | | LRW | | | Complementary | Yes for high risk women  Falling through the gaps of antenatal care  Trade off - burden of tests (physical, practical) & information gained  Fear and anxiety in pregnancy in general | *“Yes, if they’d had a history, it’s completely different. If you’d had a history then I would feel, I would feel that it’s definitely worth it. “*(Participant 6, LR, first pregnancy)  *“I think I was really, really scared, and I don’t think necessarily I would, if it had not been offered to me, I don’t think I’d have known where to go to get that, which I think if you actually, maybe if you did have family, I know they can’t be linked and you can’t be genetic or whatever, but if you did have a family history of something, maybe that test really reassured me, really reassured me, so yes. I think it’s a good thing.”* (Participant 9, LR, first pregnancy)  *“Well I think if it was going to help, I don’t think it would be a bad thing. I think women would do it if they thought it was, if it was going to give an indication of like premature birth and then there’s something that could be done to help about it, I’m sure a lot of women wouldn’t mind having it done at all. It’s just an extra test, so I think a lot of, well when you’re pregnant you feel quite anxious and stuff anyway, particularly at the beginning. And anything you can do to feel less anxious, I think sometimes more, I don’t know.”* (Patient 16, LR, one term birth)  *“I guess I don’t know what you are getting from these test results but if you are getting really important information then definitely. I mean when you are having a baby you kind of expect… to ...you know be...they are going to see down there. Do you know what I mean? You can’t be too private when you’re having a baby. For me-yeah I would. I like to know everything is alright”* (Participant 20*,* LR, first pregnancy) |
|  |  | | LRW | | | Disagreement | LR women might be less accepting of screening | *“it might feel too much for the ordinary woman who wouldn’t expect to have medical intervention as part of a normal pregnancy“* (Participant 2, LR, recurrent first trimester miscarriages, first ongoing pregnancy)  *“it’s different with each baby isn’t it? Because I think if it was my first, I would probably have every single test because I was more nervous in my first pregnancy. But then obviously, then you’ve been through a pregnancy, so you think everything might be a bit less structured, if that’s the right way to explain it”.* (Participant 8, LR, one term birth, previous colposcopy)  *“I’m not sure everybody would say yes for a routine test for that personally. I think some people will prefer not to be messed with if they don’t have to be, and there’s no history of any problems before.”* (Participant 6, LR, first pregnancy)  *“Whereas I suppose it may have been different if I hadn’t had George just because if you’ve not been through that then I suppose people talk about going through cervical smear and that’s always a bigger deal before you’ve had kids. And so would you be so willing to go for something like that if you didn’t have to or whatever?”* (Participant 12, LR, one term birth) |
