# Supplementary material for: The acceptability of cervical electrical impedance spectroscopy within a multi-modal preterm birth screening package: a mixed methods study
Source: BMC Pregnancy Childbirth. 2022 Dec 22;22:959. doi: 10.1186/s12884-022-05202-z (PMC9783720; doi:10.1186/s12884-022-05202-z)

**Supplementary Figure 1a:**

**Distribution of pre-visit STAI-6 scores by study group**


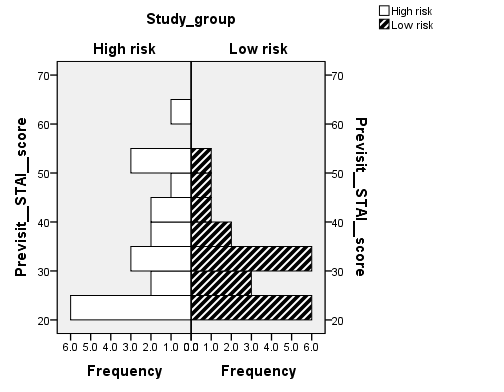


**Supplementary Figure 1b:**

**Distribution of post-visit STAI-6 scores by study group**


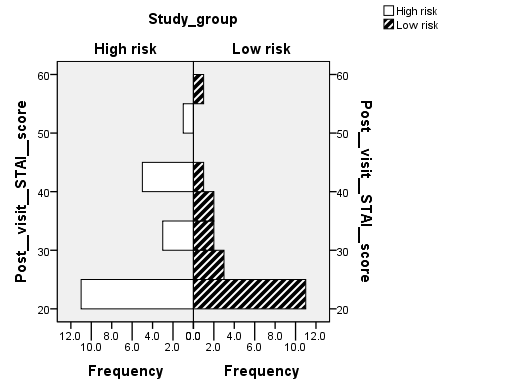

Supplement: Supplementary file 3 — Additional file 3. [file 12884_2022_5202_MOESM3_ESM.docx]
